# Technology-assisted task-sharing to bridge the treatment gap for childhood developmental disorders in rural Pakistan: an implementation science case study

**DOI:** 10.1186/s43058-022-00343-w

**Published:** 2022-09-15

**Authors:** Syed Usman Hamdani, Zill-e- Huma, Lawrence S. Wissow

**Affiliations:** 1 Global Institute of Human Development, Shifa Tameer-e-Millat University, Islamabad, Pakistan; 2 Department of Primary Care and Mental Health, University of Liverpool, Liverpool, UK; 3 Human Development Research Foundation, Rawalpindi, Pakistan; 4 University of Washington, Seattle, USA

**Keywords:** Developmental disorders, WHO mhGAP, Technology-assisted task-shifting, Family volunteers, Low-resource settings

## Abstract

**Background:**

As in many low-income countries, the treatment gap for developmental disorders in Pakistan is nearly 100%. The World Health Organization (WHO) has developed the mental Health Gap Intervention guide (mhGAP-IG) to train non-specialists in the delivery of evidence-based mental health interventions in low-resource settings. However, a key challenge to scale-up of non-specialist-delivered interventions is designing training programs that promote fidelity at scale in low-resource settings. In this case study, we report the experience of using a tablet device-based application to train non-specialist, female family volunteers in leading a group parent skills training program, culturally adapted from the mhGAP-IG, with fidelity at scale in rural community settings of Pakistan.

**Methods:**

The implementation evaluation was conducted as a part of the mhGAP-IG implementation in the pilot sub-district of Gujar Khan. Family volunteers used a technology-assisted approach to deliver the parent skills training in 15 rural Union Councils (UCs). We used the Proctor and RE-AIM frameworks in a mixed-methods design to evaluate the volunteers’ competency and fidelity to the intervention. The outcome was measured with the ENhancing Assessment of Common Therapeutic factors (ENACT), during training and program implementation. Data on other implementation outcomes including intervention dosage, acceptability, feasibility, appropriateness, and reach was collected from program trainers, family volunteers, and caregivers of children 6 months post-program implementation. Qualitative and quantitative data were analyzed using the framework and descriptive analysis, respectively.

**Results:**

We trained 36 volunteers in delivering the program using technology. All volunteers were female with a mean age of 39 (± 4.38) years. The volunteers delivered the program to 270 caregivers in group sessions with good fidelity (scored 2.5 out of 4 on each domain of the fidelity measure). More than 85% of the caregivers attended 6 or more of 9 sessions. Quantitative analysis showed high levels of acceptability, feasibility, appropriateness, and reach of the program. Qualitative results indicated that the use of tablet device-based applications, and the cultural appropriateness of the adapted intervention content, contributed to the successful implementation of the program. However, barriers faced by family volunteers like community norms and family commitments potentially limited their mobility to deliver the program and impacted the program’ reach.

**Conclusions:**

Technology can be used to train non-specialist family volunteers in delivering evidence-based intervention at scale with fidelity in low-resource settings of Pakistan. However, cultural and gender norms should be considered while involving females as volunteer lay health workers for the implementation of mental health programs in low-resource settings.

**Supplementary Information:**

The online version contains supplementary material available at 10.1186/s43058-022-00343-w.

Contributions to the literature

- The present study provides an example of using implementation science frameworks to explore the barriers to and facilitators of using technology to train non-specialist family volunteers to implement a parent skills training program and measure program fidelity at scale in a low-resource community setting.
- This study administers validated implementation science instruments to measure the program acceptability, feasibility, appropriateness, and reach and provides a comprehensive perspective of diverse stakeholders including organizations, providers, and consumers regarding program implementation in real-world settings.
- The present study suggests that although technology is a feasible and acceptable implementation strategy to train non-specialist female volunteers, several gender-related barriers—notably competing demands in their homes and community norms that potentially limit the ability of women to participate alone outside the home—could reduce the reach of the program.
- While technology-assisted application was well accepted as a training tool, most of the caregivers refused to consent to video record a live caregiver-child interaction for monitoring, quality assurance, and evaluation purposes. Understanding the use of technology from end-users’ perspectives is critical to the success of scale-up efforts using technology.

## Background

Prevalence rates of developmental disorders are estimated to be 1 in 162 children globally [1]. Many low-resource settings lack trained human resources and evidence-based intervention packages for childhood developmental disorders, resulting in a documented treatment gap of over 90% [2]. In the past two decades, there has been an increasing focus on actions to reduce this gap, particularly in low-resource settings [3]. A key landmark was the launch of the WHO Mental Health Gap Action Program (mhGAP) which provides evidence-based guidelines for the treatment of priority mental health conditions, including developmental disorders, by non-specialists in low-resource settings [4].

The mhGAP intervention guide offers a holistic, evidence-based package of care including guidelines for the assessment and management of developmental delays and disorders in children [4]. The program takes a trans-diagnostic approach, addresses a range of developmental conditions, and recommends parent skills training to promote child development using evidence-informed strategies [5]. It emphasizes training non-specialist health workers such as parents, caregivers, teachers, nurses, and community volunteers to deliver the program (task-shifting) in primary care settings or community-based facilities. Existing evidence show that mental health programs, delivered by lay healthcare workers, are found to be more effective, compared to treatment as usual to treat common mental health disorders (CMDs) in adults in low- and middle-income countries (LMICs) [6]. However, ensuring program fidelity at scale remains a key implementation challenge to disseminating such programs in low-resource settings, globally [7]. Many guidelines and models have been recently published to effectively recruit, engage, train, and supervise healthcare workers (e.g., cascade model of training and supervision) to deliver mental healthcare in the community setting. Among all these approaches, the use of technology has been identified as a complementary strategy to strengthen the task-shifting for the delivery of mental healthcare in low-resource rural settings [8].

Pakistan is a low- and middle-income country in South Asia with an estimated population of 220 million [9]. Over 6% of Pakistani children suffer from a developmental delay or disorder [10]. To address the barriers to access and improve the quality of child mental health services in low-resource settings of Pakistan, the National Ministry of Health is implementing mhGAP through primary health care platforms through task-shifting [11, 12]. The sub-district of Gujar Khan is one of the pilot sites for the implementation of mhGAP-IG in rural Pakistan. Presently, Gujar Khan has only one public special education institution, which is underfunded, oversubscribed, and inaccessible to most children with developmental disorders in need of healthcare, educational, and rehabilitative services. The nearest specialist mental health care facility is the Institute of Psychiatry, Rawalpindi, 40 km away. Previous studies conducted in Gujar Khan using the Ten Questions Screen (TQS) identified 34.2% of children as having developmental difficulties [13]. Furthermore, the lack of awareness about childhood developmental disorders in family and carers and the stigma associated with such conditions were identified as barriers that hamper access to care and increase the burden of caregiving for parents of children with developmental disorders [14]. Our earlier work in the same study settings has established that community-based female family volunteers can deliver evidence-based mental health care in community settings to bridge the mental health treatment gap [15–17].

Previously, we had converted the mhGAP guidelines into training videos for caregivers of children with developmental disorders and hosted them on a tablet device [18]. We trained non-specialist female family volunteers to deliver this technology-assisted parent skills training and pilot tested the intervention with 70 families with children with developmental disorders. This model of service delivery was found to be acceptable, feasible, and resulted in improving child outcomes [18]. Based upon these preliminary findings, we scaled up the program and evaluated its implementation to explore how technology-assisted task sharing at scale impacts training, fidelity, reach, and adoption [19]. We also explored the facilitators and barriers for scaling up technology-assisted, task-sharing interventions in low-resource rural settings [10]. Our specific research questions were (a) Can family volunteers be trained using technology to deliver parent skills training program at scale with fidelity? and (b) What is the acceptability, feasibility, appropriateness, and reach of technology-assisted, family volunteers delivered parent skills training intervention in rural community settings of Pakistan?

## Methods

### Study design

Our evaluation was part of a cluster Randomized Controlled Trial (cRCT) of the technology-assisted parent skills training intervention to improve clinical outcomes in children with developmental disorders [19]. The trial findings have been published elsewhere [10]. The evaluation used a mixed-methods design in the intervention arm of the trial. Quantitative data on implementation outcomes was collected using structured, validated measures [20] at 6 months post-program implementation. We explored participants’ perceptions about the acceptability, feasibility, appropriateness, and reach of the program and conducted qualitative in-depth interviews with program trainers, provider, and consumers at 6-months post-program implementation.

### Implementation evaluation frameworks

**Fig. 1:**
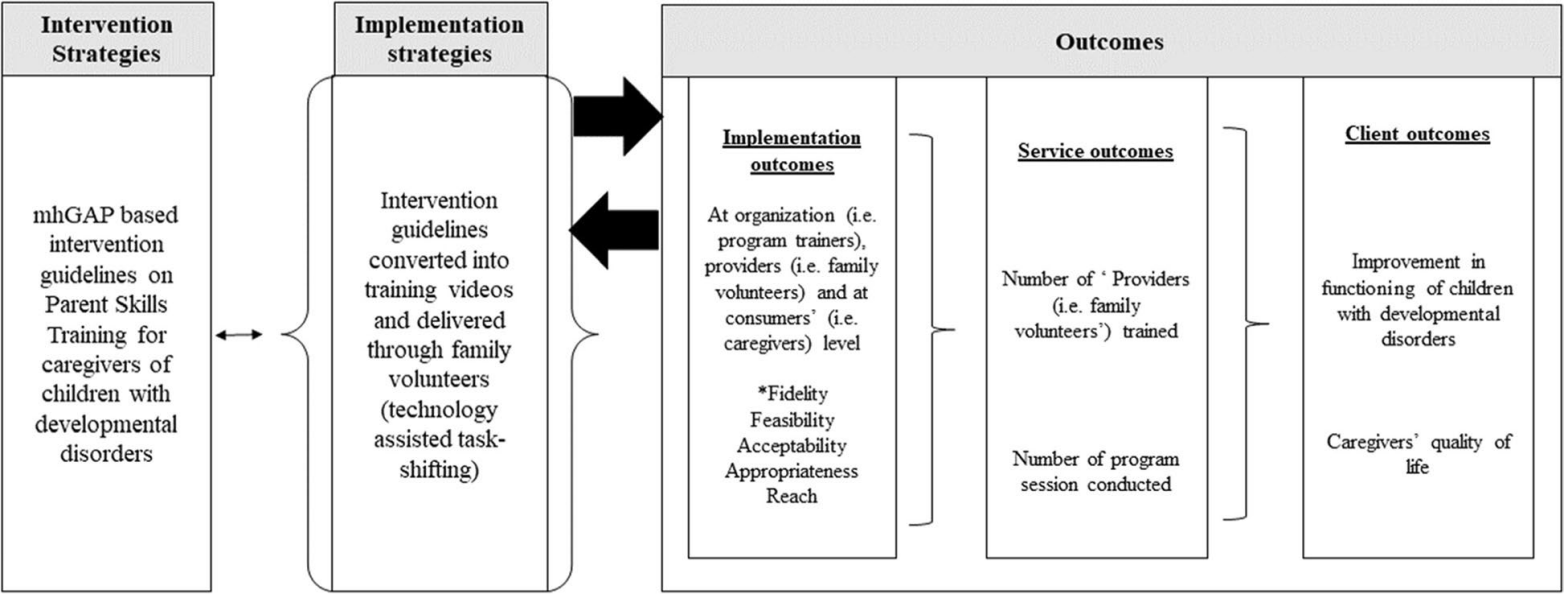
Conceptual model of implementation evaluation of technology-assisted parent skills training program delivered through “family volunteers” (adapted from Proctor et al. (2009) [13]

Our implementation evaluation was informed by Proctor and colleagues’ [21] and the RE-AIM framework [22]. According to Proctor and colleagues, evidence-based mental health interventions are implemented through strategies at different levels (organizations, providers, consumers), whose impact is interactive with implementation, service, and client outcomes (Fig. 1). This evaluative implementation science framework clearly articulates the significance of measuring implementation outcomes (such as acceptability, adoption, appropriateness, feasibility, fidelity, implementation cost, penetration, and sustainability), apart from service and client outcomes, which are usually influenced by these implementation outcomes. To complement the findings of Proctor and colleagues’ framework, we used the RE-AIM framework, which is another evaluative implementation science framework that is designed to evaluate the impact of public health interventions in real-world settings. It focuses on key program elements including its reach, effectiveness, and adoption. As the current study aimed to evaluate the implementation of technology-assisted, family volunteers delivered parent skills training at different levels, Proctor and colleagues and RE-AIM frameworks were considered more appropriate to use (instead of other implementation science process models and determinants frameworks [23]).

We measured the implementation outcomes and calculated Reach, Effectiveness, Adoption, Implementation, and Maintenance (RE-AIM) indices [24] at three levels: organization (including program trainers), family volunteers (hereafter, provider), and caregivers (hereafter, consumer). The detail on the definition of implementation outcomes and the way they have been measured in the preset study is given in Table 1. The findings on the *Effectiveness* dimension of RE-AIM, measured using a cluster randomized controlled, are published elsewhere [10]. As the implementation data in the present study was collected at 6 months post-program implementation, the *Maintenance* dimension of the RE-AIM framework (*focuses to evaluate the long-term impact of the program on clinical outcomes*) was not explored.

**Table 1:** Implementation outcomes and their description

Implementation outcome measure	Description
Fidelity	*Fidelity* is the competency of program trainers and providers to deliver the parent skills training program in the manner in which it was intended to be delivered.
Acceptability	*Acceptability* is the perception of program trainers, providers, and consumers that the program (content, mode of delivery, and delivery agent) was relevant, suitable, and satisfactory.
Feasibility	*Feasibility* is the ease of access to parent skills training program resources, participation in the training sessions, and adoption of program strategies by providers and consumers.
Appropriateness	*Appropriateness* is the perception of “program trainers, providers, and consumers” that the program is useful for its state purpose.
Reach^a^	*Reach* is the proportion of the caregivers who received the parent skills training program delivered by family volunteers.
Effectiveness^a^	*Effectiveness* is the impact of parent skills training program on child and caregivers’ clinical outcomes including child’s functioning, socio-emotional development, and caregivers’ quality of life
Adoption^a^	*Adoption* is the proportion of participants (both provider and consumers) who agreed to (i) participate and continue to implement the parent skills training program and (ii) promote/disseminate the program to other families of their community.
Implementation^a^	*Implementation* is the extent to which the parent skills training program was delivered consistently with fidelity by the providers and the cost of the program implementation.
Maintenance^a^	*Maintenance* is the long-term treatment effect of the parent skills training program on both caregivers and children (such as 2 years after the implementation of the parent skills training program)

^a^Description of RE-AIM indices (Holtrop et al. [24])

### Study settings

The program was delivered in 15 rural Union Councils (UCs) of the sub-district Gujar Khan. A UC is the smallest administrative unit within a sub-district and has a Basic Health Unit (BHU) that provides health care to the local population. Each BHU is staffed by a medical doctor, a dispenser, 15–20 Lady Health Workers (LHWs) working under the supervision of a lady health supervisor, a lady health visitor, and a vaccinator. LHWs are trained to provide preventive maternal and child health care and education in the community. Each LHW is responsible for about 100 households in her village.

### Identification of children with developmental disorders

In the present study, children with developmental disorders were identified with the help of LHWs, in two phases. Firstly, the research team along with LHWs conducted a door-to-door survey and screened all children, aged 2–12 years in the LHW’s catchment area for developmental disorders using Ten Questions Screen (TQS). Secondly, children who screened positive were assessed by a trained clinical psychologist following the clinical assessment guidelines (history and clinical examination) outlined in the mhGAP-IG [4]. A written informed consent was obtained from all the study participants before their participation in the study. Where a participant was unable to write, a thumbprint was placed in lieu of a signature.

### Identification of family volunteers

The family volunteers were family members or parents or caregivers of children with developmental disorders. They had at least eight grades of formal education and were voluntarily willing to be trained and supervised to deliver the intervention to 4–5 families in their villages for at least 6 months. The family volunteers were identified during the door-to-door screening survey. A written informed consent from each family volunteer was obtained before their participation in the study.

### Study participants

In addition to the family volunteers, the study participants for the implementation evaluation were program organizers and caregivers of children with developmental disorders.

*Program organizers* (hereafter, referred to organizers in the manuscript) were master trainers who had post-graduate education and specialized training in child mental health as well as program trainers who were graduates with master’s degrees in psychology or social sciences and had at least 1 year of experience of delivering psychological interventions in the study settings. They were trained in parent skills training program by master trainers and cascaded down the training to the providers using tablet-based tool.

*Consumers of the intervention* (caregivers of children with developmental disorders, residing in the study sub-district for the duration of the study).

### Intervention—technology-assisted, non-specialist-delivered parent skills training program

We adapted the content and delivery of the mhGAP-IG parent skills training guidelines to suit delivery by non-specialist volunteers in low-resource rural Pakistan.

#### Cultural adaptation of program content

The adapted program consists of 9-weekly group sessions and aims to train caregivers to promote communication, socioemotional development, and adaptive behaviors and manage co-morbid conditions and motor difficulties in their children. It also highlights the significance of caregivers’ self-care and teaches coping skills to caregivers to improve their own psychological well-being. A complete description of technology-assisted family volunteers delivered parent skills training program is mentioned elsewhere [10].

#### Delivery of the program

We converted the adapted program into an application consisting of training videos hosted on an Android tablet device. To help caregivers learn from the lived experiences of families of children with developmental disorders, the key program messages and strategies were converted into real-life narratives of three children with developmental disorders and their caregivers and family members. The narrative script is simple, interactive, and allows intervention providers and caregivers to discuss each scenario in the context of their own lives and develop individualized management plans for their children. To address the challenge of limited internet access in rural areas of Pakistan, we hosted the intervention videos on the tablet to make them accessible offline. The intervention resources are freely available in Urdu (the national language of Pakistan) using the following web link: https://fansforkids.org/.

#### Training and supervision of providers

**Fig. 2:**
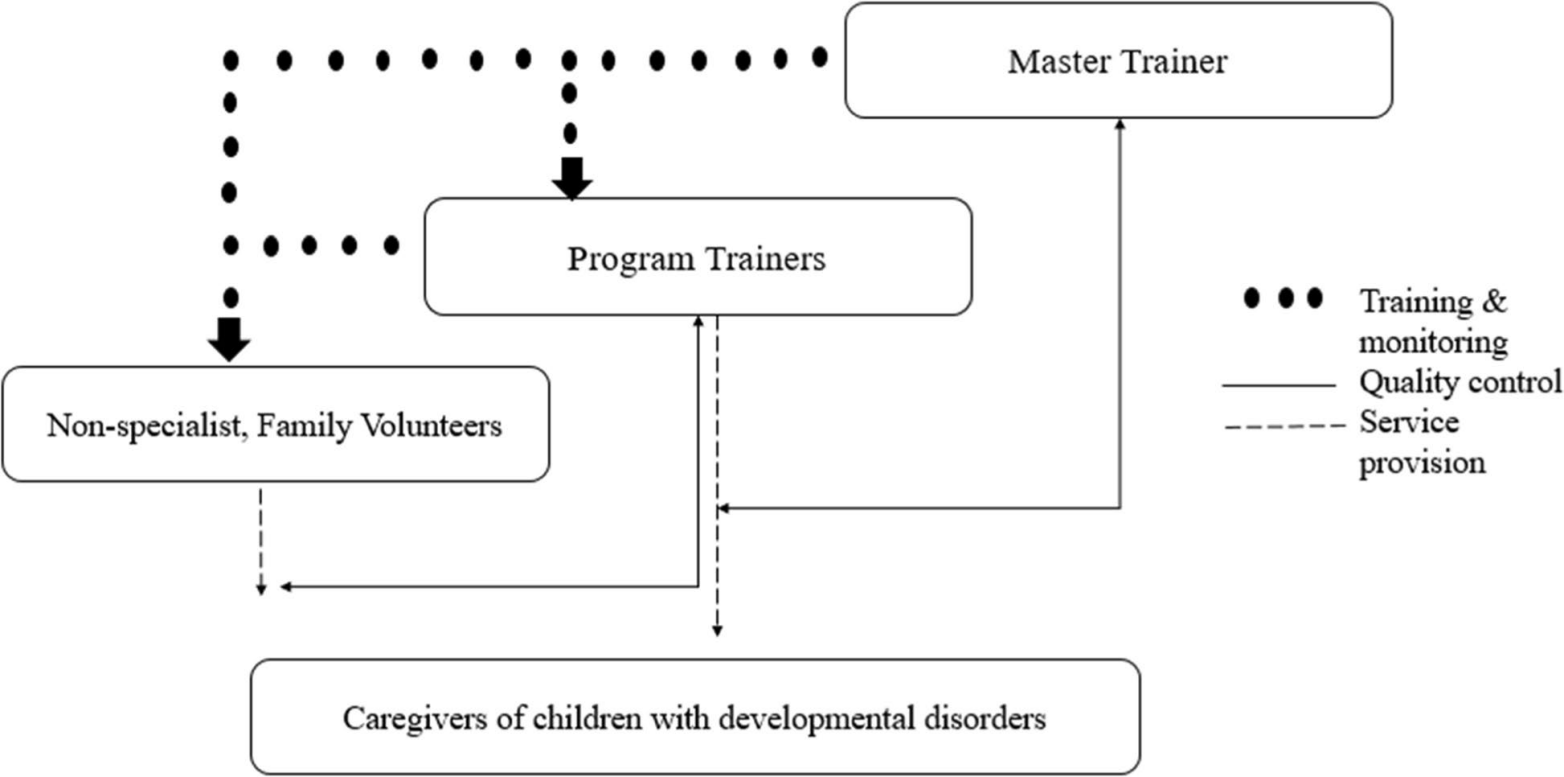
Cascaded model of training and supervision of program trainers and family volunteers (adapted from Murray et al. 2011). Note: master trainer (UH), program trainers (had at least 16 years of education and 1 year of experience in working with children and families with developmental disorders), family volunteers (FVs) (parents or caregivers of children with developmental disorders, had at least eight grades of formal education, are voluntarily willing to be trained and supervised by the trainers for at least 6 months duration of the program and cascade the training to 4–5 families in their villages)

We followed a cascade model of training and supervision [25] (Fig. 2). A master trainer trained 10 trainers who cascaded down the training to 80 potential providers, using a tablet-based training application. Each trainer was assigned a case load of 6–8 providers for training and supervision. Each trainer trained providers of the program in 9 weekly group sessions of the parent skills training program, followed by a competency rating of the providers by a master trainer on ENhancing Assessment of Common Therapeutic (ENACT) during practice sessions.

### Outcome measures

The data on implementation variables was collected during the program implementation and at 6 months post-program implementation at three implementation levels: organization, providers, and consumer (Table 2).

**Table 2:** Implementation constructs and data collection time points

Implementation outcome/construct	Organization	Providers	Consumers	During program implementation	After 6 months of program implementation
Feasibility	✓	✓	✓	–	Quantitatively/qualitatively
Acceptability	✓	✓	✓	–	Quantitatively/qualitatively
Appropriateness	✓	✓	✓	–	Quantitatively/qualitatively
Reach	✓	✓	✓	Quantitatively/qualitatively	Quantitatively
Effectiveness^a^	–	–	✓	–	Quantitatively
Adoption	–	✓	✓	–	Quantitatively
Implementation (fidelity)	–	✓	–	Quantitatively	–

Organization = program trainers; providers = family volunteers and consumers = caregivers of children with developmental disorders

^a^Effectiveness findings are published elsewhere (see Hamdani et al. [10])

### Quantitative measures and analysis

#### Measuring fidelity of the program using ENhancing Assessment of Common Therapeutic (ENACT) tool

We evaluated the fidelity of the program delivery by non-specialist providers by live rating a random sample of 20% sessions delivered by each “provider” on the ENACT tool [26], adapted for the parent skills training program. The ENACT is an observational rating scale intended to be used when observing a trainee interact with a real or standardized (mock) patient. It consists of 18 items, rated on a three-tiered response system. A score of 1 means *needs improvement*, 2 means *done partially*, and 3 means *done well*. A mean score of 2 on each domain of ENACT is required to fulfill the fidelity criteria for the program delivery. The tool has been extensively used previously to measure the common factors competence among non-specialist providers delivering mental health services in low-resource settings [27].

##### Adaptation of ENACT for parent skills training

General adaptations were made to the tool such as the use of words “parents/caregivers” and “family volunteers” instead of using “clients” and “clinician.” In the adapted ENACT, 8 items were added to reflect the essential therapeutic skills required to deliver the technology-assisted parent skills training program in a group setting. Domains of ENACT (*n* = 7) that were specific for one-on-one therapy sessions were excluded in consultation with the developer of ENACT. The final adapted ENACT for parent skills training consisted of a 20-item tool and incorporated a four-tiered response system. A mean score of 2.5 on each domain of adapted ENACT was required to fulfill the fidelity criteria for the program delivery (see supplementary material).

#### Intervention monitoring data

Apart from collecting data on the fidelity of intervention delivery by non-specialists, we also collected data on intervention dosage and analyzed notes of the supervision meetings.

#### Implementation outcome measures

We used the Applied Mental Health Research Dissemination and Implementation measurements (AMHR D&I) [20] to measure the implementation outcomes at the organization, provider, and consumer levels. These tools have been validated to measure the implementation outcomes including feasibility, acceptability, appropriateness, adoption, and penetration of the mental health programs in low- and middle-income countries [20]. We translated and adapted the tools following the standard procedure (forward and back translation of tool, conducting cognitive interviews and piloting of the tool and blind back translation) for translation and adaptation of the mental health instruments for trans-cultural research [28].

##### Adaptation of AMHR D&I measures for parent skills training program

General adaptations: The general adaptations to the AMHR D&I measures included the change of the program’s name, replacing the word “provider” with “family volunteers” and “mental health services” to “services for caregivers of children with developmental disorders.” As the parent skills training program targets the whole family and has been designed for children of both genders with developmental disorders and their caregivers, the items exploring the appropriateness of the program from a gender perspective such as *does program fit with the male/female culture in your country?* were removed from all three versions (consumer provider, and organization). The reach sub-scale assesses the reach of mental health services/programs to underprivileged groups, males/females, orphan children, and people in the military office. All such items related to the reach of the program to male/female and military personnel were excluded as these items were contextually and conceptually not relevant.

##### Specific adaptations

At the provider’s level, the intervention-specific examples such as the delivery of the program, monitoring of the child’s progress using tablets, and participation in monthly supervision meetings were included. In the current study, the providers became family volunteers due to altruism and because of their intrinsic needs of helping their community members or helping their own extended family members (baradari) to improve their health outcomes. As all-female family volunteers were delivering the intervention voluntarily, neither any official contract was issued to them by the implementation organization nor they were employed somewhere else during their participation in the study (80% were not currently employed at the time of enrollment); therefore, the whole domain of “individual professionalism” that was designed to evaluate *(1) how does their current role, being the providers of mental health program, and help community health providers feel successful in their job; (2) allow them to have job stability; (3) is aligned with their professional goals; and (4) create more opportunities for their career advancement*, was excluded in an adapted version of the tool; each item is scored on a 4-point scale ranging from 0 *not at all* to 3 *a lot*, with an additional category for *do not know/not applicable*. We calculated Cronbach’s alpha for each domain of adapted tools to assess the reliability. The values of Cronbach’s alpha ranged from 0.70 to 0.90, except for the domain of “reach” (*α* = 0.30).

##### Quantitative analysis

The quantitative data was analyzed using descriptive analysis. To compare the implementation variables across three levels of participants (organization, providers, and consumers), the mean differences in each domain of AMHR D&I measures were calculated.

### Qualitative measures and analysis

We conducted 30 in-depth interviews with six groups of participants (see Table 3 for categories of participants) at 6 months post-program implementation using a semi-structured interview guide. The respondents were selected purposively depending upon their willingness and availability. The average duration of each interview was 55 min (± 9.5). All interviews were audio recorded and transcribed verbatim.

**Table 3:** Sample matrix for in-depth qualitative interviews (*N* = 30)

Categories of participants	*N* = 30
Providers who completed the parent skills training program and delivered the intervention to caregivers	5
Providers who completed the parent skills training program but did not deliver the intervention to caregivers	5
Providers who did not complete the training in the parent skills training program	5
Consumers who completed the training in parent skills training intervention	5
Consumers who did not complete the parent skills training intervention	5
Organizers (trainers) of parent skills training program who trained providers in the parent skills training intervention	5

#### Qualitative analysis

The data were analyzed using the framework analysis [29]. In the first step, the transcriptions of interviews were read again and again for familiarization, identification of any data (thoughts, feelings, and impressions) that seemed of potential interest and significant for our research question, and to generate codes. After generating the initial coding of few transcriptions using an inductive approach, an analytical framework around a priori categories of interest (acceptability, feasibility of the program) was developed to index the data from the coded transcriptions. After indexing the data of all transcriptions, the research team summarized the indexed data for each category and organized it in a chart form. Finally, mapping and interpretation of the data were done to describe each category, identify any commonalities and differences between the data (across the different groups of participants), and map the connections between categories to explore causality (*if there was any*).

## Results

The case study has been reported following the Standards for Reporting Implementation Studies (StaRI) checklist [30] (please see additional file).

### Parent volunteer (provider) participation and characteristics

Out of 62 trained providers, 36 delivered the program to caregivers in local villages. Twenty-six providers were not able to deliver the program due to health problems (*n* = 7), competing family commitments (*n* = 7), having to work (*n* = 6), or not achieving competency (*n* = 6).

The mean age of the 36 participating providers was 35 (± 4.38) years. For 61% of the providers, education level was 10 years (Table 4). All 36 providers were females (95% mothers and 5% paternal aunts). Eighty-one percent of them were not currently employed, and 70% were living in the nuclear family system.

**Table 4:** Demographic details of participants who provided quantitative data

Variables	Consumers (*n* = 166)	Providers (*n* = 36)	Organization (*n* = 14)
Age (mean, SD), years	35 (±7.6)	35 (±4.38)	25.5 (±1.8)
Education			
Uneducated	44 (26.5%)	–	–
Primary—grade 5	39 (23.5%)	6 (17%)	–
Middle—grade 8	17 (10.2%)	4 (11%)	–
Matriculate—grade 10	35 (21.1%)	22 (61%)	–
College and university—grades 11–16	31 (18.6%)	4 (11%)	14 (100%)
Number of sessions attended (mean, SD)	–	9 (0.00)	–
Number of sessions delivered (mean, SD)	–	7.60 (±2.31)	–

### Parent (consumer) participation and characteristics

**Fig. 3:**
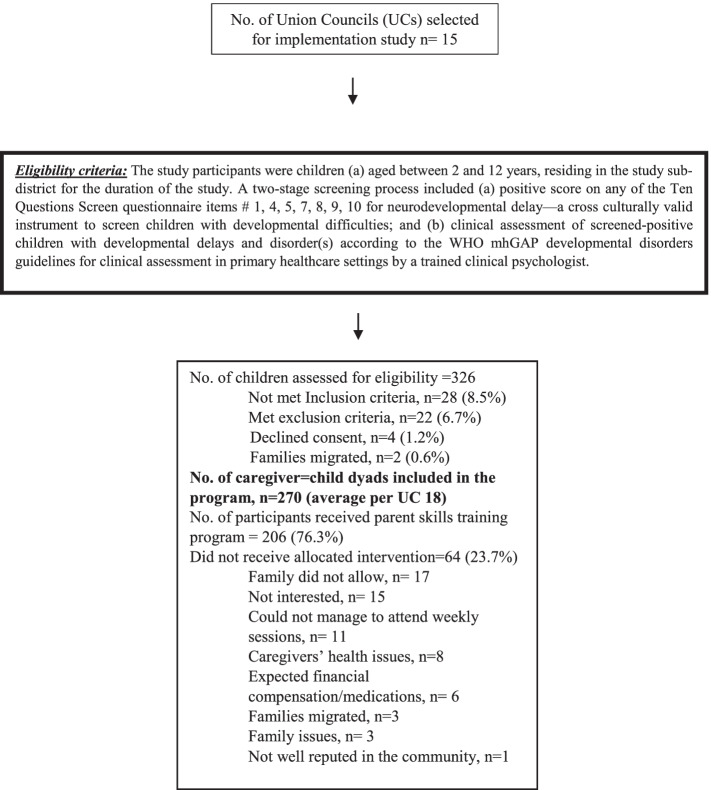
Flow of participants through the study

Out of 326 potential parent-child dyads in 15 Union Council, 270 parents of children with development disorders who met the eligibility criteria were enrolled in the study (*see* Fig. 3*for the eligibility criteria and flow of the participants through the study*). Out of 270 caregivers enrolled, 85% (230/270) of the trial participants attended 6 sessions (± 1), and only 166 caregivers participated in the implementation evaluation. The mean age of the consumers was 35 (± 7.6) years. Half of the consumers had less than 5 years of education or no education (50%, 83/166).

### Provider delivery of the program

A total of 504 sessions of the program were delivered by 36 providers in 79 groups within the duration of 6 months. The average group size was 6 (range from 5 to 7). Eighty-five percent (230 /270) of the consumers attended 6 sessions (range from 5 to 7). The average duration of a group session was 102 min (± 15). The average group size was 6 (range from 3 to 7).

### Cascading supervision sessions with providers

Over the period of 6 months, 10 program trainers organized 30 group supervision meetings of providers. The average attendance in the group supervision was 75%. In turn, providers conducted 178 group supervision sessions with consumers after initial training of 9 sessions. The attendance in these supervision sessions was 73%.

### Fidelity of the program

To assess the fidelity of the program, 20% of the sessions (103/504) spread over 36 providers were rated at three time points (between program sessions 1–3, 4–6, and 7–9). Cronbach’s alpha reliability of the adapted version of ENACT for all three-time points ranged between 0.70 and 0.93. The intervention sessions were delivered with good fidelity as all providers scored on an average of 2.5 or more (mean [SD], 2.97 ± 0.21) out of 4 on all items of the adapted ENACT at the three time points.

The primary clinical outcome of interest was change in the child’s functioning at 6 months post-intervention implementation. The results of the overall trial have been published elsewhere [10].

### Acceptability, feasibility, appropriateness, and reach of the program at scale

The data on implementation outcomes was collected from 166 consumers, 36 providers, and 14 members of the program team including program trainers (Table 5).

**Table 5:** Descriptive statistics of AMHR D&I* measures (at consumer, provider, and organizer levels)

Scale	No. of items	M (SD)	Observed range	Possible range on instrument	*α*
Consumer’s level (*n* = 166)					
Acceptability	15	39.49 (5.92)	15–45	0–45	0.91
Feasibility	13	31.29 (4.76)	13–38	0–39	0.76
Appropriateness	10	24.62 (4.17)	9–30	0–30	0.82
Reach	4	6.99 (1.98)	3–12	0–12	0.37
Provider’s level (*n* = 36)					
Acceptability	9	34.2 (3.01)	18–34	9–36	0.89
Feasibility	15	49.7 (5.73)	26–45	15–60	0.95
Appropriateness	9	31.8 (4.62)	14–33	9–36	0.85
Reach	4	10.6 (2.01)	4–11	4–16	0.75
Organizer’s level (*n* = 14)					
Acceptability	5	17.79 (2.45)	12–20	5–20	0.87
Feasibility	12	42.29 (5.44)	30–48	12–48	0.90
Appropriateness	10	36.43 (4.16)	26–40	10–40	0.88
Reach	4	11.07 (2.84)	6–15	4–16	0.77

*AMHR D&I Applied Mental Health Research Dissemination and Implementation

#### Acceptability of the program

Caregivers (*n* = 166) indicated that the program was highly acceptable to them (mean scores of acceptability domain, 39.49 ± 5.92). They reported high satisfaction on receiving the program training from a “family volunteer” (item response *a lot* 2.77 ± 0.47) as she was perceived to be someone who listens, understands, and addresses their questions or concerns about their child and program (2.74 ± 0.49) and takes interest in their concerns and problems (2.75 ± 0.49). Family volunteers were able to earn the trust of the caregivers of children with developmental disorders (2.77 ± 0.46) as she was perceived as qualified enough to deliver the program (2.77 ± 0.45).

The program was perceived as highly acceptable by the providers (*n* = 36) (mean scores of acceptability domain, 34.2 ± 3.01). They not only liked the program materials (3.90 ± 0.31), but they also felt good while delivering the program to the families (3.70 ± 0.67). They felt the program was an appropriate intervention (3.90 ± 0.31); moreover, the skills they learnt while delivering the program were perceived to be useful for the families (3.90 ± 0.31). They were also satisfied with the amount of the training (3.90 ± 0.31) and supervision (3.60 ± 0.51) they received by the program trainers.

The program acceptability at the organization level was very high (17.79 ± 2.45). The organizers felt that the delivery of the program was a source of contentment for the program team and trainers. It was not only helpful and beneficial for the reputation of the organization, but it could also create more opportunities of service delivery for the organization.

#### Feasibility of the program

The caregivers perceived that participating in the parent skills training program was feasible for them (31.29 ± 4.76). However, they reported challenges in taking time out of their daily routine to attend weekly training sessions (1.86 ± 0.90): in managing household responsibilities, in implementing program strategies at home by themselves (2.15 ± 0.82), and in finding childcare for their other children during the time they were attending the session (2.09 ± 1.03). Caregivers reported that community members did talk negative about the families seeking program services for their children (1.80 ± 0.75).

The program was very feasible for providers to deliver (domain score 49.7 ± 5.73). The item-wise analysis showed that they felt skilled enough to deliver the program (3.80 ± 0.422) and had appropriate time to implement all the activities of the program (3.40 ± 0.84) including organizing group, providing training and peer supervision to caregivers in their villages and continuously monitoring child’s progress during program implementation. Although providers in the current study were delivering services on volunteer basis and they were only given a tablet device (*an intervention delivery aid*) for the duration of the program; being enrolled in the program as community workers positively contributed to enhance their skills and confidence. This finding is consistent with the results of previous studies on evaluating the social impact of doing community work on community healthcare workers in Pakistan. Studies show that even with the minimalistic stipend of rupees 2500 (PKR), the program had a positive impact on their well-being and contributed to women empowerment, perhaps by increasing their access to healthcare resources, enhancing their decision-making skills, and opening employment opportunities [31].

The program trainers perceived that implementing the parent skills training program was feasible for the organization (42.29 ± 5.44). According to them, the organization had sufficient resources including a sufficient pool of trainers (3.86 ± 0.53), sufficient finances (2.75 ± 0.75), an adequate transportation system (3.57 ± 0.75), and the necessary equipment (3.86 ± 0.36) to train non-specialists and implement the program in the study settings. Moreover, program trainers also mentioned that they received adequate administrative support (3.62 ± 0.65) and clinical supervision (3.77 ± 0.43) to provide supervision and support to the providers.

#### Appropriateness of the program

As the intervention content was adapted into a training application with “real-life” narratives [18] both the consumers and providers rated the program as highly appropriate and in keeping with the local traditions and cultural values (the mean score of consumers on the appropriateness domain was 24.62 ± 4.17 and 31.8 ± 4.62 for providers). The consumers perceived that the program was a good way to address their and their child’s problems (2.43 ± 0.71). The program strategies they learned helped them to deal with their worries (2.46 ± 0.63) and solve their problems related to their children (2.49 ± 0.78). The consumers agreed that these strategies would also be useful to other caregivers of their community who have similar problems (2.43 ± 0.66). However, attending group training sessions would be inconvenient for these caregivers due to their household responsibilities (1.72 ± 0.89).

Providers reported that they were satisfied with their role as a provider of the program, and they will keep implementing the program to the families (3.50 ± 0.85) in their villages under the supervision of the trainers (3.20 ± 1.1). They appreciated the idea of implementing the program through non-specialist, family volunteers; however, *difficulties in seeking permission from their own family to deliver program in the community and competing demands on time due to household responsibilities* were identified as potential barriers to continue to implement the program through family volunteers. Previous studies conducted in Pakistan on exploring the factors affecting the work experience of community health workers in rural settings of Pakistan show that providing door-to-door healthcare services in the community settings, especially paying visits to the houses of “non-kin” [32] is not considered prestigious [33–35], and because of this, they face disapproval from the community [35]. They also face family pressure in connection with their work often due to a common misconception that because of their work, family reputation is at-risk [33]. Thus, ensuring the involvement of family members, particularly effectively taking male family members on board, becomes imperative to increase family support, ensure women’s safety while field visits, and increase the appropriateness of the “role of provider” overall.

The program trainers perceived that the program was highly appropriate to the needs and cultural values of the consumers (mean scores of the domain, 36.43 ± 4.16). According to the program trainers, as the program content was designed to meet the needs of the consumers, it was likely to be useful to manage the problems of children with developmental disorders and their caregivers. They perceived that the delivery of the program to the families of children with developmental disorders in the study area was aligned with the core values and goals of their organization and implementing this program was a priority for leaders at their organization.

#### Reach of the program

Both the program trainers and providers perceived that the reach of the program was high (mean scores of reach domain for program trainers and providers were 11.07 ± 2.84 and 10.6 ± 2.01, respectively). The item level analysis indicated that according to the providers, the community members were fully aware that the program was being delivered in the community by the family volunteers (3.50 ± 0.70). They also perceived that all those caregivers in their community who might be in need of a parent skills training program, specifically parents from low socio-economic status, would still find this program useful and attend the training (2.40 ± 0.69). However, according to the consumers the reach of the program was comparatively low (6.99 ± 1.98).

We also calculated the reach of the program using RE-AIM indices. We successfully established 36 potentially self-sustaining village-based “family networks” led by 36 trained providers working under the supervision of trainers from the local nongovernmental organization. The target population for the program was 270 caregivers and their children who met the eligibility criteria and were enrolled in the program. Out of 270 participants, 85% (230/270) of the population received the program training by 36 providers using the technology platform. Although the reach of the program during the implementation phase was high (*might be due to program acceptability*), however, various barriers, such as family pressure and restricted women’s mobility in the rural settings of Pakistan, can inhibit the scale-up implementation of the program.

### Qualitative findings

Most of the respondents in the qualitative study reported that the program content addressed the problems of children with developmental disorders and caregivers’ concerns that they faced in taking care of their child (see Table 6 for themes and relevant quotes). The use of a training application hosted on the tablet was appreciated by the respondents. The participants also mentioned that program illustrations (personas, scenarios, and sittings) were quite reflective of their surrounding environment, people, and cultural norms and helped them to effectively learn program strategies. The qualitative data analysis indicated that family support from their own family was identified as an important factor in caregivers’ involvement with the program. However, the engagement of providers with the program relied, in turn, on the support they received from their own family members in delivering the program. Moreover, the household responsibilities such as looking after the children and domestic animals and working in the fields got in the participants’ way to attend the program sessions. For both the providers and consumers, there was reliance on their own family members to cover other household responsibilities so that they had time to participate in the program sessions. Family-related obligations have been frequently reported as one of the significant barriers in the participation of the task-sharing mental health interventions previously [36]. “Domestic work” has been identified as one of the major perceived barriers encountered by community healthcare workers in the delivery of services (Khan, 2012). The continuation of their work depends on how well they manage their work and household responsibilities. This is perhaps normatively in Pakistan; women are expected to perform household chores rather than involving in economic activities outside their homes [32]. Often community healthcare workers received support to perform household responsibilities by other women of their family, particularly in the joint family system, however, primary responsibility of their home, especially the responsibility of upbringing of their children lies with them [33]. Therefore, giving due considerations to the socio-cultural context in which female family volunteers operate is important for sustainable delivery of the program in community settings.

**Table 6:** Findings of qualitative interviews

Objectives	Themes	Quote (source)
Acceptability to consumers	Increase in knowledge	*We learnt how to deal with children and (particularly) their behavioral issues. We also came to know about why do they (children with developmental disorders) (mis)behave; it was very nice to learn about this program because we did not know about these simple but helpful strategies that we know now.* (Consumer)
	Trustable relationship with the provider	*We find it easy to talk to her (provider) and discuss our challenges (of implementing program strategies) with her during the sessions. She patiently listens to our other (family) problems that are not related to the child (and program) and shows interest; we feel good after sharing our problems with her.* (Consumer)
Acceptability to providers	Acceptance from family and community	*My family has supported me when I shared with them that trainers will come to my place (to do the trainings of other family volunteers). My husband fully supported me (and generously offered) that the mothers of other children who have problem can come to our house for participating in the program.* (Family volunteer, who completed the training and delivered the program)
	Facilitated learning through the use of training videos	*Use of tablet and videos is a good way when mothers cannot understand explanation of the trainer, they can understand it in a better way through training videos.* (Family volunteer, who completed the training and delivered the program)
	Group sessions—created a sense of shared experience	*We learned from experiences of each other and realized that we were always feeling alone (in this problem) but there are others who have similar problem with their children. There were many mothers in our group with different problems of their children and we came to know about their problems also.* (Family volunteer, who completed the training but did not deliver the program)
Feasibility to consumers	Household responsibilities as a barrier	*We all have household and community responsibilities such as working in the fields, taking care of animals and fetching water; if one has a child with special needs which demands special attention then it sometimes become too much for one to handle.* (Consumer)
Feasibility to provider	Timing and duration of group sessions	*Most mothers were very co-operative. They came to my place on-time and attended the training sessions attentively. If someone had to go back to home for some urgent work, we would catch-up at a later time. It was also a good learning experience for all the mothers in my village.* (Family volunteer)
	Cooperation from caregivers	*It was easy for people who were educated (to understand the content of program) but it was difficult for the people who were illiterate to understand (the content of program). After completing the training session using videos and explanation from the trainer, the content was easily understood by the illiterate mothers as well.* (Family volunteer, who completed training and delivered the program)
Appropriateness to consumers	Cultural relevance of intervention content	*In our village we do the same. We have Charpoy (Cot) where we all sit together, get sun bath, some children play with toy cars and others ride the cycles around. So, whatever shown in the video was similar to surroundings of our village. It was very much similar… It was not about city environment. It was mostly about village and our children.* (Consumer)
Appropriateness to provider	Relevance of intervention strategies with problems of children	*The problems that were showed through video by tablet were very much similar to the problems of children in our village and most of the people could relate to the problems. It includes speech problems, like some children also have difficulty in moving around, difficulty in changing position at bed.* (Family volunteer, who completed the training and delivered the program)

Both providers and consumers expressed their satisfaction over the duration of the sessions and suggested to schedule the sessions a week apart so that consumers are able to attend the sessions conveniently. The respondents expressed their satisfaction on organizing the trainings at basic health units (located within their respective villages) or at the house of providers. Intervention delivery in village-based groups was regarded as an acceptable format of parent skills training by a number of respondents. According to participants, sitting together as a group served as a learning platform for them in which they could share their problems with each other. The respondents expressed that having a session in a group helped them to validate and normalize their feelings about their child’s health condition; additionally, they helped to learn from each other through this experience.

## Discussion

This case study describes the implementation evaluation of a family volunteer-delivered, technology-assisted parent skills training program for children with developmental disorders in low-income rural communities. The results showed that technology can be used to train female family volunteers in the delivery of parent skills training program at scale with fidelity. The findings of quantitative and qualitative analyses show that the program’s content, mode of delivery using technology, and non-specialist family volunteers as the delivery agent were perceived to be highly acceptable, feasible, and appropriate to consumers, providers, and organizers, and the technology-assisted program had a high reach according to them. The study also illustrated several other important points about scaling up task-shifting interventions in low-resource settings. First, in addition to the tablet platform, adapting materials to make them engaging and readily identifiable as commonly encountered situations in the community was a key factor in the acceptability of the program. Second, measuring the competency at the end of the training was an essential step in assuring fidelity; nearly 10% of family volunteers entering the training could not be deployed to their communities because they did not achieve the required level of skill.

While the technology-assisted training and delivery were feasible and well-accepted, other barriers—notably competing demands in their homes faced by the family volunteers and the parents they worked with, plus community norms that potentially limited the ability of women to participate alone outside the home—reduced the reach of the program. The use of task-shifting approaches to deliver psychological interventions at a doorstep via community-based female healthcare workforce has been frequently used previously and found to be an acceptable implementation strategy [15, 17, 37, 38]. However, numerous individuals, family, intra-household dynamics, and sociocultural challenges are faced by female lay healthcare workers that affect their participation in program delivery. Especially in more conservative and patriarchal communities, barriers faced by female healthcare workers include but are not limited to; lack of autonomy, adequate experience, and education; restrictions from in-laws and husbands to participate and deliver the program [39]; and lack of transport, gender dynamics (reluctance of family members to let females travel alone or at their own), and mental health stigma [40]. The lack of adequate training and support from mental health professionals is yet another obstacle faced by female healthcare workers in other similar settings [41, 42]. Moreover, sociocultural norms have been reported as another most significant barrier to the provision of mental health services using task-shifting approaches in low- and middle-income countries (LMICs) [36]. Much importance has been given to consider the cultural norms in the intervention design and delivery to increase the program’s acceptability. Future versions of the program may need to address these factors, perhaps by involving additional family members or providing other support to the female volunteers such as providing monetary rewards during program administration and ensuring support of male family members. Finally, despite rating the program very positively, consumers reported difficulty carrying out what they had learned at home and required more supportive supervision to learn the strategy and time to practice these strategies.

In the present study, we trained family volunteers to deliver parent skills training program to the caregivers of children with developmental disorders in their own villages. The findings of qualitative in-depth interviews showed that locally identified family volunteers were highly acceptable as delivery agents. According to the participants, family volunteers exhibited characteristics of trustworthiness and altruism which contributed significantly to increase the program’s acceptability and made it appropriate for the targeted community/population. In Pakistan, as in many other countries, women/mothers are children’s main caretakers. Based on formative work [18] and to be consistent with social norms in Pakistan, we used females as volunteers to deliver the program. This was also consistent with the idea that peer-to-peer support can be most effective when the beneficiary sees the peer supporter as knowledgeable but also with similar gender [43]. Also, studies show that the use of culturally responsive interventions and effective engagement with local stakeholders positively contribute to the increase acceptance of the task-sharing programs [36]. Task-shifting has been frequently used as an effective implementation strategy to tackle the shortage of healthcare workforce, especially in low-resource settings, where there is a scarcity of mental health professionals [44, 45]. Delivery of trans-diagnostic, skills training interventions through non-specialist family facilitators is a widely accepted approach and has the potential to deliver psychosocial interventions in low-resource, fragile, and humanitarian settings [46].

However, one of the frequent concerns regarding the implementation of evidence-based practices by non-specialist at scale is the decrease in program fidelity and its effectiveness [7] due to the diverse mental health needs of the populations and challenges associated with maintaining the quality of training and supervision of non-specialists at scale. Mobile-based devices and services have been frequently used previously for data collection, training, and delivery of healthcare services by community providers and are promising strategies to improve healthcare delivery in LMICs [47]. In the present study, we addressed these challenges by developing a tablet-based application for training and program implementation. This innovation is consistent with the current evidence that highlights the considerable potential of digital mental health interventions to maintain high fidelity of the evidence-based programs at scale [44] and are reported to be as good as training by specialists [46]. The findings of qualitative analysis showed that the use of culturally appropriate avatars and real-life stories of three children with developmental disorders and their families helped providers and caregivers to gain a deeper understanding of the issues faced by children and learn strategies and skills to manage child’s conditions. This is consistent with the findings of previous studies in which digital mental health interventions were reported to be highly feasible and acceptable to not only promote but also to treat mental health problems in low-resource settings [48, 49]. Also, careful sensitization and adaptation of mHealth interventions according to socio and cultural factors can facilitate to achieve its intended positive outcome of the innovation [50]. The findings of our study are critical to the efforts of promoting the use of technology platforms and training non-specialists to bridge the treatment gap for mental health in low resource settings, globally.

A key hallmark of our study is the use of implementation science frameworks and valid and reliable implementation outcome measures alongside a clinical trial to measure the effectiveness of the intervention [51], which are often overlooked in implementation science studies [52, 53]. Furthermore, we translated and culturally adapted the implementation outcome measures following the standard procedure recommended for translation and adaptation of the mental health instruments for trans-cultural research [54]. Although our study was not powered to assess the implementation effectiveness, we systematically collected the data on implementation outcome measures at consumer, provider, and organization levels. The use of implementation science frameworks and mixed-methods study design to collect data helped us to identify critical insights that are imperative for the successful implementation of parent skills training program in low-resource community settings of Pakistan. Our study highlighted that technology in the form of training videos can be a feasible and acceptable strategy to deliver mental health program with fidelity at scale in real-world settings.

The definitive randomized evaluation of intervention effectiveness demonstrated that the program did not result in statistically significant improvement in the clinical outcomes of children when implemented at scale. This difference in the intervention impact from the exploratory study [18] could be because of multiple reasons; in the definitive cRCT, we used trans-diagnostic eligibility criteria to enroll children with developmental disorders in the cRCT; as a result, children with a range of developmental disorders conditions including intellectual disability, motor difficulties, speech and communication difficulties, and Down syndrome were included in the study. Although the effectiveness evaluation was powered to detect improvement in child’s functioning, the heterogeneous nature of the study sample might have led to inadequate power to evaluate a statistically significant improvement in children with different developmental conditions and severity. Moreover, a relatively short duration of the intervention (i.e., 6 months) might not have been adequate to improve clinical outcomes in children with complex and long-standing unmet developmental needs. The parent skills intervention resulted in improved quality of life of caregivers of children with developmental disorders [10]. However, we were not able to establish whether improved parental health-related quality of life translated into enhancing their competency to interact and engage with the child due to the high refusal rate to video record mother-child interaction. Moreover, the data from implementation outcomes offered some important explanations about the difficulties that parents reported in using the skills at home and the barriers to participating in the full number of sessions, which might have created hindrances for caregivers to effectively learn the intervention strategies and implement these with their children at home. As Ilozumba et al. identified that a number of contextual factors including household responsibilities, financial issues, transportation, availability of services, autonomy and family influence, and relationship between healthcare providers and participants play an important role in the adoption and adherence of technology-based interventions and to bring its desired positive change [55]. It highlights a need of providing more hands-on support to the caregivers to implement the intervention strategies in real-life settings, apart from training them in skills training program only.

While our study offers detailed insight into the feasibility and acceptability of using technology-assisted task-shifting strategies to bridge the treatment gap for developmental disorders in low-resource settings, it has a number of limitations. Although we used psychometrically strong implementation outcome measures, our study was not powered to evaluate the implementation effectiveness. The study was not designed and powered to evaluate the implementation outcomes (acceptability, feasibility, and appropriateness) as potential mediators to change clinical outcomes (change in caregivers’ quality of life) in caregivers. Moreover, the data on the implementation outcome was collected at only one time point. The data collection at multiple time points can help to explore whether the fidelity, acceptability, feasibility, and appropriateness of the program were sustained over time. In addition to that, the use of implementation science frameworks helped us to systematically plan and execute the implementation of parent skills training program in real-world settings and to unpack implementation processes at the organization, provider, and consumer levels which are often poorly understood and missing in a traditional randomized evaluation.

## Conclusions

Technology-enhanced training of volunteers has many positive features, but it needs to be deployed in a culturally congruent way including understanding the way in which the volunteers’ participation potentially challenges their traditional roles in families and communities to improve program implementation and increase its reach. Moreover, context-specific evaluation of the program using implementation outcome measures and frameworks, embedded within the traditional RCTs could be beneficial to identify the priority challenges that need urgent mitigation before further scale up of the program in real-world settings. Such trials are warranted to (a) compile and consolidate implementation data using validated implementation outcome measures that can be used or adapted for a variety of settings and implemented with a range of stakeholders, (b) evaluate the adoption and integration of evidence-based intervention strategies in the healthcare system; (c) evaluate the impact of improved implementation on clinical outcomes, and (d) measure the sustainability of such community-based initiatives over the period of time.

## Supplementary Information

**Additional file 1.** Supplementary information.

**Additional file 2.** Standards for Reporting Implementation Studies: the StaRI checklist.

## Data Availability

The dataset generated and analyzed during the current study is available from the corresponding author upon reasonable request.

## Abbreviations

WHO: World Health Organization
mhGAP-IG: Mental Health Gap Action Program - Intervention Guide
RE-AIM: Reach, Evaluation, Adoption, Implementation, and Maintenance
UC: Union Council
ENACT: ENhancing Assessment of Common Therapeutic factors
BHU: Basic health unit
TQS: Ten Questions Screen
cRCT: Cluster randomized controlled trial
LHW: Lady health worker
FVs: Family volunteers
AMHR D&I: Applied Mental Health Research Dissemination and Implementation
AMD: Adjusted mean difference
CI: Confidence interval

## References

[1] Elsabbagh M, Divan G, Koh Y-J, Kim YS, Kauchali S, Marcín C (2012). Global prevalence of autism and other pervasive developmental disorders. Autism Res.

[2] Patel V, Kieling C, Maulik PK, Divan G (2013). Improving access to care for children with mental disorders: a global perspective. Arch Dis Child.

[3] Collins PY, Pringle B, Alexander C, Darmstadt GL, Heymann J, Huebner G (2017). Global services and support for children with developmental delays and disabilities: bridging research and policy gaps. PLoS Med.

[4] WHO (2016). mhGAP intervention guide for mental, neurological and substance use disorders in non-specialized health settings: Mental Health Gap Action Programme (mhGAP): version 2.0.

[5] Reichow B, Servili C, Yasamy MT, Barbui C, Saxena S (2013). Non-specialist psychosocial interventions for children and adolescents with intellectual disability or lower-functioning autism spectrum disorders: a systematic review. PLoS Med.

[6] van Ginneken N, Tharyan P, Lewin S, Rao GN, Meera SM, Pian J, Chandrashekar S, Patel V. Nonspecialist health worker interventions for the care of mental, neurological and substance-abuse disorders in lowand middle-income countries. Cochrane Database Syst Rev. 2013;(11):CD009149. 10.1002/14651858.CD009149.pub2. Update in: Cochrane Database Syst Rev. 2021 Aug 5;8:CD009149. 10.1002/14651858.CD009149.pub2 24249541

[7] Chambers DA, Glasgow RE, Stange KC (2013). The dynamic sustainability framework: addressing the paradox of sustainment amid ongoing change. Implement Sci.

[8] Hoeft TJ, Fortney JC, Patel V, Unützer J (2018). Task-sharing approaches to improve mental health care in rural and other low-resource settings: a systematic review. J Rural Health.

[9] World Population Review. Pakistan population - 2022 2022 [cited 2022 May 17]. Available from: https://worldpopulationreview.com/countries/pakistan-population.

[10] Hamdani SU, Huma ZE, Suleman N, Akhtar P, Nazir H, Masood A (2021). Effectiveness of a technology-assisted, family volunteers delivered, brief, multicomponent parents’ skills training intervention for children with developmental disorders in rural Pakistan: a cluster randomized controlled trial. Int J Ment Heal Syst.

[11] Najam S, Chachar AS, Mian A (2019). The mhGAP; will it bridge the mental health treatment gap in Pakistan?. Pak J Neurol Sci.

[12] NHS R&C. WHO Mental Health Global Action Programme (mhGAP): situation analysis of primary healthcare system in Pakistan. NHS R&C. Available from https://phkh.nhsrc.pk/sites/default/files/2019-06/Mental%20Health%20Global%20Action%20Programme%20%28mhGAP%29%20Report%202016.pdf.

[13] Mirza I, Mehmood T, Tareen A, Davidson L, Rahman A (2008). Feasibility study on the use of the ten questions screen by lady health workers to detect developmental disabilities in Pakistan. JPPS..

[14] Hamdani SU, Atif N, Tariq M, Minhas FA, Iqbal Z, Rahman A (2014). Family networks to improve outcomes in children with intellectual and developmental disorders: a qualitative study. Int J Ment Heal Syst.

[15] Rahman A, Malik A, Sikander S, Roberts C, Creed F (2008). Cognitive behaviour therapy-based intervention by community health workers for mothers with depression and their infants in rural Pakistan: a cluster-randomised controlled trial. Lancet.

[16] Sikander S, Ahmad I, Atif N, Zaidi A, Vanobberghen F, Weiss HA (2019). Delivering the Thinking Healthy Programme for perinatal depression through volunteer peers: a cluster randomised controlled trial in Pakistan. Lancet Psychiatry.

[17] Atif N, Lovell K, Husain N, Sikander S, Patel V, Rahman A (2016). Barefoot therapists: barriers and facilitators to delivering maternal mental health care through peer volunteers in Pakistan: a qualitative study. Int J Ment Heal Syst.

[18] Hamdani S, Minhas FA, Iqbal Z, Rahman A (2015). Model for service delivery for developmental disorders in low-income countries. Pediatrics..

[19] Hamdani SU, Akhtar P, Zill EH, Nazir H, Minhas FA, Sikander S (2017). WHO Parents Skills Training (PST) programme for children with developmental disorders and delays delivered by family volunteers in rural Pakistan: study protocol for effectiveness implementation hybrid cluster randomized controlled trial. Global Ment Health (Camb).

[20] Haroz E, Bolton P, Nguyen A, Lee C, Bogdanov S, Bass J (2019). Measuring implementation in global mental health: validation of a pragmatic implementation science measure in eastern Ukraine using an experimental vignette design. BMC Health Serv Res.

[21] Proctor E, Silmere H, Raghavan R, Hovmand P, Aarons G, Bunger A (2011). Outcomes for implementation research: conceptual distinctions, measurement challenges, and research agenda. Adm Policy Ment Health Ment Health Serv Res.

[22] Glasgow RE, Vogt TM, Boles SM (1999). Evaluating the public health impact of health promotion interventions: the RE-AIM framework. Am J Public Health.

[23] Nilsen P (2015). Making sense of implementation theories, models and frameworks. Implement Sci.

[24] Holtrop JS, Estabrooks PA, Gaglio B, Harden SM, Kessler RS, King DK (2021). Understanding and applying the RE-AIM framework: clarifications and resources. J Clin Transl Sci.

[25] Murray LK, Dorsey S, Bolton P, Jordans MJ, Rahman A, Bass J (2011). Building capacity in mental health interventions in low resource countries: an apprenticeship model for training local providers. Int J Ment Heal Syst.

[26] Kohrt BA, Jordans MJ, Rai S, Shrestha P, Luitel NP, Ramaiya MK (2015). Therapist competence in global mental health: development of the ENhancing Assessment of Common Therapeutic factors (ENACT) rating scale. Behav Res Ther.

[27] Kohrt BA, Mutamba BB, Luitel NP, Gwaikolo W, Onyango Mangen P, Nakku J (2018). How competent are non-specialists trained to integrate mental health services in primary care? Global health perspectives from Uganda, Liberia, and Nepal. Int Rev Psychiatry.

[28] Rahman A, Iqbal Z, Waheed W, Hussain N (2003). Translation and cultural adaptation of health questionnaires. J Pak Med Assoc.

[29] Gale NK, Heath G, Cameron E, Rashid S, Redwood S (2013). Using the framework method for the analysis of qualitative data in multi-disciplinary health research. BMC Med Res Methodol.

[30] Pinnock H, Barwick M, Carpenter CR, Eldridge S, Grandes G, Griffiths CJ (2017). Standards for Reporting Implementation Studies (StaRI) Statement. BMJ (Clinical research ed).

[31] Khan A (2011). Lady health workers and social change in Pakistan. Econ Polit Wkly.

[32] Jejeebhoy SJ, Sathar ZA. Women’s Autonomy in India and Pakistan: Influence of Religion and Region. Population and Development Review. 2001;27(4):687–712.

[33] Mumtaz Z, Salway S, Waseem M, Umer N (2003). Gender-based barriers to primary health care provision in Pakistan: the experience of female providers. Health Policy Plan.

[34] Khan A (1999). Mobility of women and access to health and family planning services in Pakistan. Reprod Health Matters.

[35] Mumtaz Z, Salway S, Nykiforuk C, Bhatti A, Ataullahjan A, Ayyalasomayajula B (1982). The role of social geography on lady health workers’ mobility and effectiveness in Pakistan. Soc Sci Med.

[36] Le PD, Eschliman EL, Grivel MM, Tang J, Cho YG, Yang X (2022). Barriers and facilitators to implementation of evidence-based task-sharing mental health interventions in low- and middle-income countries: a systematic review using implementation science frameworks. Implement Sci.

[37] Cooper PJ, Tomlinson M, Swartz L, Landman M, Molteno C, Stein A (2009). Improving quality of mother-infant relationship and infant attachment in socioeconomically deprived community in South Africa: randomised controlled trial. BMJ (Clinical research ed).

[38] Ali BS, Rahbar MH, Naeem S, Gul A, Mubeen S, Iqbal A (2003). The effectiveness of counseling on anxiety and depression by minimally trained counselors: a randomized controlled trial. Am J Psychother.

[39] Ahmed J, Ur Rehman S, Shahab M (2017). Community midwives’ acceptability in their communities: A qualitative study from two provinces of Pakistan. Midwifery..

[40] Khan AW, Amjad CM, Hafeez A, Shareef R (2012). Perceived individual and community barriers in the provision of family planning services by lady health workers in Tehsil Gujar Khan. J Pak Med Assoc.

[41] Shahmalak U, Blakemore A, Waheed MW, Waheed W (2019). The experiences of lay health workers trained in task-shifting psychological interventions: a qualitative systematic review. Int J Ment Heal Syst.

[42] Steege R, Taegtmeyer M, McCollum R, Hawkins K, Ormel H, Kok M (1982). How do gender relations affect the working lives of close to community health service providers? Empirical research, a review and conceptual framework. Soc Sci Med.

[43] Feldhaus I, Silverman M, LeFevre AE, Mpembeni R, Mosha I, Chitama D (2015). Equally able, but unequally accepted: gender differentials and experiences of community health volunteers promoting maternal, newborn, and child health in Morogoro Region, Tanzania. Int J Equity Health.

[44] Bass JK, Hamdani SU, Stein DJ, Bass JK, Hofmann SG (2019). 4 - Scaling up and implementing psychotherapies in low-resource settings. Global Mental Health and Psychotherapy.

[45] Rahman A, Hamdani SU, Siller MML (2018). Supporting intervention providers and families in South Asia. Handbook of parent-implemented interventions for very young children with autism.

[46] Rahman A, Akhtar P, Hamdani SU, Atif N, Nazir H, Uddin I, Nisar A, Huma Z, Maselko J, Sikander S, Zafar S. Using technology to scale-up training and supervision of community health workers in the psychosocial management of perinatal depression: a non-inferiority, randomized controlled trial. Glob Ment Health (Camb). 2019;6:e8. 10.1017/gmh.2019.7. 10.1017/gmh.2019.7 PMC6533850 31157115

[47] Agarwal S, Perry HB, Long LA, Labrique AB (2015). Evidence on feasibility and effective use of mHealth strategies by frontline health workers in developing countries: systematic review. Trop Med Int Health.

[48] Chandra PS, Sowmya HR, Mehrotra S, Duggal M (2014). ‘SMS’ for mental health—feasibility and acceptability of using text messages for mental health promotion among young women from urban low income settings in India. Asian J Psychiatr.

[49] Carrasco A. Acceptability of an adventure video game in the treatment of female adolescents with symptoms of depression. Res Psychother Psychopathol Process Outcome. 2016;19:10–18. 10.4081/ripppo.2016.182.

[50] Mengesha W, Steege R, Kea AZ, Theobald S, Datiko DG (2018). Can mHealth improve timeliness and quality of health data collected and used by health extension workers in rural Southern Ethiopia?. J Public Health (Oxford, England).

[51] Bauer MS, Damschroder L, Hagedorn H, Smith J, Kilbourne AM (2015). An introduction to implementation science for the non-specialist. BMC Psychol.

[52] Cook DA, Beckman TJ (2009). Does scale length matter? A comparison of nine- versus five-point rating scales for the mini-CEX. Adv Health Sci Educ Theory Pract.

[53] Lewis CC, Fischer S, Weiner BJ, Stanick C, Kim M, Martinez RG (2015). Outcomes for implementation science: an enhanced systematic review of instruments using evidence-based rating criteria. Implement Sci.

[54] Wild D, Grove A, Martin M, Eremenco S, McElroy S, Verjee-Lorenz A (2005). Principles of good practice for the translation and cultural adaptation process for patient-reported outcomes (PRO) measures: report of the ISPOR Task Force for Translation and Cultural Adaptation. Value Health.

[55] Ilozumba O, Dieleman M, Kraamwinkel N, Van Belle S, Chaudoury M, Broerse JEW (2018). “I am not telling. The mobile is telling”: factors influencing the outcomes of a community health worker mHealth intervention in India. PLoS One.

