# Supplementary material for: Technology-assisted task-sharing to bridge the treatment gap for childhood developmental disorders in rural Pakistan: an implementation science case study
Source: Implement Sci Commun. 2022 Sep 15;3:99. doi: 10.1186/s43058-022-00343-w (PMC9479305; doi:10.1186/s43058-022-00343-w)
Supplement: Supplementary file 1 — Additional file 1. Supplementary information. [file 43058_2022_343_MOESM1_ESM.docx]

“Lessons from the field: case studies in global implementation science”

This submission is part of a collection of global implementation science case studies that have been commissioned by the Fogarty International Center (FIC) at the U.S. National Institutes of Health, in collaboration with partner NIH Institutes and Centers and key external partner organizations. The aims of this collection are to: (1) demonstrate the rigorous application of implementation science in the context of global health (2) highlight the use and adaptation of frameworks, implementation strategies, research designs, and measures for LMIC settings and (3) assess the utility and impact of implementation science in these settings. The Center for Global Health Studies (CGHS) at FIC found that there does not appear to be one cohesive collection of implementation science case studies, especially one relating to global health and LMICs that spans different organizations, countries, or diseases. The cases also highlight the rigor and **process** of doing implementation science in the global field, as well as describing practical considerations and challenges. CGHS intends to publish 8-10 cases from different geographic areas with diverse disease foci. The project is guided by a steering committee chaired by Dr. Rohit Ramaswamy at the Cincinnati Children's Hospital Medical Center and made up of 14 implementation science experts with broad expertise. For reference the Case Study Protocol used by the authors in conjunction with the journal formatting guidelines is found below.

Thank you.

Global Implementation Science Case Studies for Health Framework

*Fogarty International Center*

*Center for Global Health Studies (CGHS)*

The first objective of this collection of case studies will be to demonstrate the rigorous application of implementation science in the context of global health. The second objective is to highlight the actual use and adaptation of frameworks, implementation strategies, research designs, and measures for LMIC settings. The final objective is to assess the utility and impact of implementation science in these settings. For the purposes of this collection of case studies **implementation science** will be defined as “the study of methods to promote the adoption and integration of evidence-based practices, interventions and policies into routine health care and public health settings” (Fic.nih.gov).

Each case study will address one or more of the implementation outcomes defined in **Appendix 1**. These were adapted from *Proctor et al. 2011*. Cases will span diverse disease areas/conditions/settings and represent different geographic regions. All cases will focus on research that has been completed, while acknowledging implications for future research or data and outcomes, where applicable.

**Audience**

Potential, new, or existing global implementation researchers, public health practitioners, policy makers, funding organizations, program implementers, and students are the audience. Educators of implementation science may wish to use or adapt these cases as a teaching resource.

**Word Limit**

5,500 (not including supplemental files) following the *Implementation Science* [**research article format**](https://implementationscience.biomedcentral.com/submission-guidelines/preparing-your-manuscript/research). To maintain the prescribed word limit, sections of the Framework may be included as an Appendix or supplemental material at the discretion of the case study authors.

**Components**

**All numbered main components are required (Abstract, contributions to the literature, background, methods, results, discussion, and conclusions).**

**Note that not every subcomponent may be relevant to a particular case study and may be excluded at the discretion of the authors. Include the subcomponents that are applicable. Elements within a section should be addressed in the order most conducive to the narrative of the case study.**

**1. Abstract**

The abstract should not exceed 350 words. Please minimize the use of abbreviations and do not cite references in the abstract. Reports of randomized controlled trials should follow the CONSORT extension for abstracts. Include trial registration at the end of abstract, if applicable. The abstract must include the following separate sections:

Background - the context and purpose of the study

Methods - how the study was performed, and statistical tests used

Results - the main findings

Conclusions - brief summary and potential implications

**2. Contributions to the Literature**

Per the journal requirements, all manuscripts submitted to *Implementation Science* must include a bulleted statement describing what the paper, if published, would add to the literature in implementation science. The statement should consist of three to five bullet points of no more than 100 words in total. Authors should not simply restate their findings or conclusions; the statement must contextualize the paper in the full implementation science literature and provide a succinct statement about what it adds. The statement should be in lay language and understandable to all readers, written for readers of moderate English literacy.

**3. Background**

**Context**

1) A description of the implementation problem and the IS specific questions the research seeks to answer 2) The health condition/disease focus and the related global burden 3) An overview of the geographic location & setting (health department, healthcare setting, school, workplace, places of worship, other community settings) of the research and 4) The study design of the research.

**4. Methods**

**Frameworks for Implementation**

1) The desired IS outcomes and why they were selected 3) Identification and justification of the IS model or framework applied 4) Explanation of how the model or framework was operationalized and if this was effective. For example, was the model/framework used to stage the project, to identify appropriate measures, or to assess progress, etc. and 5) Address if these models or frameworks were adopted retrospectively to evaluate the implementation process or proactively to guide the implementation process.

**Intervention, Adaption and Delivery**

Defined according to the NIH D&I Working Group codebook as: “Any program, policy, or guideline intended to improve health that has been tested and demonstrated to be effective in a particular context. This is opposed to an implementation strategy per se.”

1) An overview of the intervention 2) Justification for the intervention and the evidence base supporting its effectiveness 3) The target population for the intervention 4) How the intervention was delivered and by whom 5) How the framework/model was adapted to the cultural context.

**Implementation Strategies**

1) A description of the IS strategies that were selected/developed/applied using the following guidelines: <https://implementationscience.biomedcentral.com/articles/10.1186/1748-5908-8-139> (If relevant to a specific study design, use appropriate StaRI criteria checklist or SQUIRE checklist for quality improvement); 2) How the strategies were selected and implemented; 3) How strategies were adapted; 4) The measured implementation outcomes of the applied strategies; and 5) Specific targets for each strategy if identified.

**Measurement**

1) The indicators and instruments used; 2) Were these validated instruments; 3) Were they adapted or translated; 3) Time points of data collection and their justification; 4) Sample size calculations; and 5) Analysis plan, type of analysis and power to detect any difference in implementation outcomes.

**Design**

1) The study design used; 2) The contingencies made to account for choice junctures; 3) Were contingency plans needed and how were designs adapted; and 4) What changes were made to the study protocol (If relevant to a specific study design, use appropriate StaRI criteria checklist).

**Stakeholder Engagement**

1) A description of the key stakeholders, including decision-makers and program implementers; 2) How and when were they engaged throughout the research; and 3) How stakeholder engagement was assessed.

**5. Results**

1) The relevant implementation results or measures *(for complete list see Appendix 1);* 2) Relevant implementation process outcomes; 3) Any links between implementation and health outcomes; and 4) How implementation quality was evaluated.

**6. Discussion**

**Impact**

1) An assessment of the utility and impact of implementation science in this setting, including from a health equity perspective when appropriate; 2) The dissemination strategies used effectively; and 3) How dissemination strategies were tailored or adapted to specific audience segments.

**Ethics**

1) The ethical challenges that arose (Brownson et al. 2018); 2) Any human subjects involved in the research; 3) Who should/can provide informed consent; 4) If equipoise is necessary; 5) How scientific rigor can be protected in real-world settings and 6) How ethical challenges were addressed.

**Sustainability**

1) How was sustainability conceptualized and planned for in regard to the implemented intervention(s); 2) Identify and examine any factors that enhanced or hindered the sustainability of the intervention during or after implementation; 3) How was sustainability measured and assessed.

**7. Conclusions**

1) Limitations and challenges; 2) General insights on the use of implementation science in global health; and 3) Next steps for this body of work.

**Appendix 1: Implementation Science Outcomes for Reference**

a. Acceptability - is the perception among implementation stakeholders that a given treatment, service, practice, or innovation is agreeable, palatable, or satisfactory.

b. Adoption - the intention, initial decision, or action to try or employ an innovation or evidence-based practice. Adoption also may be referred to as “uptake.”

c. Appropriateness - the perceived fit, relevance, or compatibility of the innovation or evidence-based practice for a given practice setting, provider, or consumer; and/or perceived fit of the innovation to address a particular issue or problem.

d. Feasibility - the extent to which a new treatment, or an innovation, can be successfully used or carried out within a given agency or setting.

e. Fidelity - the degree to which an intervention was implemented as it was prescribed in the original protocol or as it was intended by the program developers.

f. Implementation cost - the cost impact of an implementation effort.

g. Penetration - the integration of a practice within a service setting and its subsystems.

h. Sustainment - the extent to which a newly implemented treatment is maintained or institutionalized within a service setting’s ongoing, stable operations.

i. Scale/Spread – the degree to which an intervention is expanded to new populations or delivery systems. This extends to multiple service settings and multiple subsystems and cuts across organizational/geographic boundaries.

j. Settings readiness to adopt – evaluate the settings readiness, capacity, or appetite for change.
